# Supplementary material for: Estimating the health impact of nicotine exposure by dissecting the effects of nicotine versus non-nicotine constituents of tobacco smoke: A multivariable Mendelian randomisation study
Source: PLoS Genet. 2024 Feb 9;20(2):e1011157. doi: 10.1371/journal.pgen.1011157 (PMC10883537; doi:10.1371/journal.pgen.1011157)
Supplement: S1 Note — (DOCX) [file pgen.1011157.s001.docx]

**S1 Note**

These results are also shown in S2 Fig, S2 Table, and S3 Table.

***CHD***

Among current smokers, there is no clear evidence to suggest that either NMR or smoking heaviness affect CHD risk (OR = 1.01, 95% CI 0.95 to 1.07, OR = 1.10 95% CI 0.89 to 1.36 respectively), nor is there evidence to suggest a clear effect of NMR or smoking heaviness in the IVW-MVMR analysis (OR = 0.99, 95% CI 0.95 to 1.05, OR = 1.27 95% CI 0.89 to 1.82 respectively). There is considerable evidence of heterogeneity in theses analyses, and some evidence of horizontal pleiotropy or bias due to population stratification in the analysis among never smokers, but there is no clear evidence to suggest directional pleiotropy and the results are supported by the MR-Egger analyses.

***COPD***

The MR-IVW results indicate that increased NMR and smoking heaviness increase the risk of developing COPD among current smokers (OR = 1.37, 95% CI 1.20 to 1.56; OR = 13.41, 95% CI 7.53 to 23.88 respectively). However, the MVMR-IVW results indicate a protective effect of nicotine exposure when smoking heaviness is accounted for (NMR OR = 1.15, 95% CI 1.02 to 1.31) and a stronger effect of smoking heaviness when NMR was accounted for among current smokers (OR = 16.90 95% CI 6.82 to 41.84). These results are supported by the MR-Egger and MVMR-Egger results and there is no clear evidence of heterogeneity or directional pleiotropy or horizontal pleiotropy or bias due to population stratification among never smokers (where precise null effects are observed).
