# Supplementary material for: Estimating the health impact of nicotine exposure by dissecting the effects of nicotine versus non-nicotine constituents of tobacco smoke: A multivariable Mendelian randomisation study
Source: PLoS Genet. 2024 Feb 9;20(2):e1011157. doi: 10.1371/journal.pgen.1011157 (PMC10883537; doi:10.1371/journal.pgen.1011157)
Supplement: S2 Note — (DOCX) [file pgen.1011157.s002.docx]

**S2 Note**

Buchwald and colleagues [1] reported summary-level statistics from a genome-wide association study (GWAS) meta-analysis of cotinine plus 3’hydroxycotinine (COT+3HC). Single nucleotide polymorphisms (SNPs) were reported as independent if they explained additional variance in a step-wise conditional regression using genome-wide complex trait analysis [2]. The reported data is available on request from the authors. Ware and colleagues [3] report summary-level statistics from a GWAS meta‑analysis of cotinine levels (per standard deviation change) among 4,548 daily smokers of European Ancestry (data available at: <https://doi.org/10.5523/bris.182rhz19hg3lz1172a7yfcap9v>). SNPs were reported as independent if they reached genome-wide significance using an iterative process of conditional analyses. For both cotinine and COT+3HC, there were a limited number of independent SNPs available in the outcome datasets, so a relaxed threshold (p < 5 x 10^-6^) was used to identify independent SNPs for inclusion in the analysis.

**References**

1. Buchwald J, Chenoweth MJ, Palviainen T, Zhu G, Benner C, Gordon S, et al. Genome-wide association meta-analysis of nicotine metabolism and cigarette consumption measures in smokers of European descent. Mol Psychiatry. 2020. Epub 2020/03/12. doi: 10.1038/s41380-020-0702-z. PubMed PMID: 32157176; PubMed Central PMCID: PMCPMC7483250.

2. Yang J, Lee SH, Goddard ME, Visscher PM. GCTA: a tool for genome-wide complex trait analysis. Am J Hum Genet. 2011;88(1):76-82. Epub 2010/12/17. doi: 10.1016/j.ajhg.2010.11.011. PubMed PMID: 21167468.

3. Ware JJ, Chen X, Vink J, Loukola A, Minica C, Pool R, et al. Genome-Wide Meta-Analysis of Cotinine Levels in Cigarette Smokers Identifies Locus at 4q13.2. Sci Rep. 2016;6:20092. Epub 2016/02/03. doi: 10.1038/srep20092. PubMed PMID: 26833182; PubMed Central PMCID: PMCPMC4735517.
