# Supplementary material for: Estimating the health impact of nicotine exposure by dissecting the effects of nicotine versus non-nicotine constituents of tobacco smoke: A multivariable Mendelian randomisation study
Source: PLoS Genet. 2024 Feb 9;20(2):e1011157. doi: 10.1371/journal.pgen.1011157 (PMC10883537; doi:10.1371/journal.pgen.1011157)
Supplement: S3 Note — (DOCX) [file pgen.1011157.s003.docx]

**S3 Note**

In UK Biobank, 49,979 individuals were genotyped using the UK BiLEVE array and 438,398 using the UK Biobank axiom array. Pre-imputation QC, phasing and imputation are described elsewhere [1]. In brief, prior to phasing, multiallelic SNPs or those with minor allele frequency (MAF) ≤1% were removed. Phasing of genotype data was performed using a modified version of the SHAPEIT2 algorithm [2]. Genotype imputation to a reference set combining the UK10K haplotype and HRC reference panels [3] was performed using IMPUTE2 algorithms [4]. The analyses presented here were restricted to autosomal variants within the HRC site list using a graded filtering with varying imputation quality for different allele frequency ranges. Therefore, rarer genetic variants are required to have a higher imputation INFO score (Info>0.3 for MAF >3%; Info>0.6 for MAF 1-3%; Info>0.8 for MAF 0.5-1%; Info>0.9 for MAF 0.1-0.5%) with MAF and Info scores having been recalculated on an in-house derived ‘European’ subset [5].

Individuals with sex-mismatch (derived by comparing genetic sex and reported sex) or individuals with sex-chromosome aneuploidy were excluded from the analysis (n=814). We restricted the sample to individuals of ‘European’ ancestry as defined by an in-house k-means cluster analysis performed using the first 4 principal components provided by UK Biobank using R software environment. The largest cluster from the in-house k-means cluster analysis was n=464,708. To model population structure in the sample, we used 143,006 directly genotyped SNPs, obtained after filtering on MAF > 0.01; genotyping rate > 0.015; Hardy-Weinberg equilibrium p-value < 0.0001 and LD pruning to an r2 threshold of 0.1 using PLINKv2.00.

**References**

1. Bycroft C, Freeman C, Petkova D, Band G, Elliott LT, Sharp K, et al. The UK Biobank resource with deep phenotyping and genomic data. Nature. 2018;562(7726):203-9. Epub 2018/10/12. doi: 10.1038/s41586-018-0579-z. PubMed PMID: 30305743; PubMed Central PMCID: PMCPMC6786975.

2. O'Connell J, Sharp K, Shrine N, Wain L, Hall I, Tobin M, et al. Haplotype estimation for biobank-scale data sets. Nat Genet. 2016;48(7):817-20. doi: 10.1038/ng.3583.

3. Huang J, Howie B, McCarthy S, Memari Y, Walter K, Min JL, et al. Improved imputation of low-frequency and rare variants using the UK10K haplotype reference panel. Nature Communications. 2015;6(1):8111. doi: 10.1038/ncomms9111.

4. Howie B, Marchini J, Stephens M. Genotype imputation with thousands of genomes. G3 (Bethesda). 2011;1(6):457-70. Epub 2012/03/03. doi: 10.1534/g3.111.001198. PubMed PMID: 22384356; PubMed Central PMCID: PMCPMC3276165.

5. Mitchell R, Hemani G, Dudding T, Corbin L, Harrison S, Paternoster L. UK Biobank Genetic Data: MRC-IEU Quality Control, version 2 - Datasets - data.bris. databris. 2018. doi: doi:10.5523/bris.1ovaau5sxunp2cv8rcy88688v.
