# Supplementary material for: Estimating the health impact of nicotine exposure by dissecting the effects of nicotine versus non-nicotine constituents of tobacco smoke: A multivariable Mendelian randomisation study
Source: PLoS Genet. 2024 Feb 9;20(2):e1011157. doi: 10.1371/journal.pgen.1011157 (PMC10883537; doi:10.1371/journal.pgen.1011157)
Supplement: S4 Note — (DOCX) [file pgen.1011157.s004.docx]

**S4 Note**

We identified Chronic Obstructive Pulmonary Disease (COPD) cases as participants who responded that they had been diagnosed with COPD in response to the question "Has a doctor ever told you that you have had any of the conditions below?".

Forced expiratory volume in 1 second (FEV-1) and forced vital capacity (FVC) were measured multiple times using a Vitalograph spirometer. We used the ‘best measure’ of both FEV-1 and FVC which were identified as the highest value recorded with no measurement issues reported.

Coronary heart disease (CHD) diagnosis was determined using linked hospital admission data with ICD codes relating to ischemic heart disease (ICD-9 410-414; ICD-9 4100-4149; ICD-10 I20-I25). The measure included angina pectoris, acute myocardial infarction, subsequent myocardial infarction, certain current complications following acute myocardial infarction, other acute ischaemic heart diseases, and chronic ischaemic heart disease.

Heart rate (beats per minute) was assessed on multiple occasions per session. Heart rate can be affected by numerous factors such as exercise [1] and stress [2]. To allow time for the participant’s heart rate to normalise during the session, we used the second measure taken within the session.

***UK Biobank health outcome field codes***

Field IDs can be entered into the online variable search platform where information can be found on measurement (available at <http://biobank.ndph.ox.ac.uk/showcase/search.cgi>).

Chronic Obstructive Pulmonary Disease: 22130

Forced Expiratory Volume and Forced Vital Capacity: 20150 and 20151

Coronary Heart Disease: 41270 and 41271

Heart rate: 102

**References**

1. Evans DL. Cardiovascular adaptations to exercise and training. Vet Clin North Am Equine Pract. 1985;1(3):513-31. Epub 1985/12/01. doi: 10.1016/s0749-0739(17)30748-4. PubMed PMID: 3877552.

2. Kim H-G, Cheon E-J, Bai D-S, Lee YH, Koo B-H. Stress and Heart Rate Variability: A Meta-Analysis and Review of the Literature. Psychiatry investigation. 2018;15(3):235-45. Epub 2018/02/28. doi: 10.30773/pi.2017.08.17. PubMed PMID: 29486547.
