# Supplementary material for: Estimating the health impact of nicotine exposure by dissecting the effects of nicotine versus non-nicotine constituents of tobacco smoke: A multivariable Mendelian randomisation study
Source: PLoS Genet. 2024 Feb 9;20(2):e1011157. doi: 10.1371/journal.pgen.1011157 (PMC10883537; doi:10.1371/journal.pgen.1011157)
Supplement: S5 Note — (DOCX) [file pgen.1011157.s005.docx]

**S5 Note**

While harmonising the exposure datasets with the outcome datasets, two SNPs associated with cigarettes per day (CPD) were removed due to having intermediate allele frequencies (rs1737894, rs28438420). Two SNPs were associated with both NMR and CPD (rs56113850, rs117824460), and one SNP associated with NMR was not available in the CPD GWAS dataset (rs34638591). The p-value for the rs117090198 SNP-exposure effect size was unusually high prior to the conditional analysis in the original GWAS [1] and the original authors could not provide a definitive explanation for this. We removed this SNP to reduce heterogeneity. Where SNPs associated with the nicotine metabolite ratio (NMR) or CPD were not available in either of the two other datasets, we searched for proxy SNPs with a minimum linkage disequilibrium (LD) R^2^ of 0.8. In MVMR analyses, all SNPs included in the model should be independent of each other (i.e., the SNPs associated with NMR must also be independent of the SNPs associated with CPD and vice versa). To ensure overall independence, we clumped the full list of SNPs (N = 59 SNPs, LD R^2^ < 0.1, clumping window > 500 kb). Given the limited number of SNPs associated with the NMR, SNPs associated with CPD were dropped from the analysis rather than SNPs associated with NMR to preserve instrument strength (i.e., no NMR SNPs were dropped during the clumping stage).

**References**

1. Buchwald J, Chenoweth MJ, Palviainen T, Zhu G, Benner C, Gordon S, et al. Genome-wide association meta-analysis of nicotine metabolism and cigarette consumption measures in smokers of European descent. Mol Psychiatry. 2020. Epub 2020/03/12. doi: 10.1038/s41380-020-0702-z. PubMed PMID: 32157176; PubMed Central PMCID: PMCPMC7483250.
