# Supplementary material for: Estimating the health impact of nicotine exposure by dissecting the effects of nicotine versus non-nicotine constituents of tobacco smoke: A multivariable Mendelian randomisation study
Source: PLoS Genet. 2024 Feb 9;20(2):e1011157. doi: 10.1371/journal.pgen.1011157 (PMC10883537; doi:10.1371/journal.pgen.1011157)
Supplement: S6 Note — (DOCX) [file pgen.1011157.s006.docx]

**S6 Note**

We used four complimentary univariable Mendelian randomisation (MR) methods (inverse variance weighted [IVW], MR-Egger, weighted median-based estimation and weighted modal-based estimation) [1-4]. Using a variety of Mendelian randomisation (MR) methods with different assumptions with respect to horizontal pleiotropy – which occurs when single genetic variants influence multiple phenotypes [5]– allows us to better understand whether the effect of our exposure on our outcome is causal. Consistent results across these methods provide stronger evidence to support a true causal effect which is not the result of a false positive [6]. We also estimated the weighted regression dilution (I^2^_GX_) for each MR-Egger analysis [7] and applied simulation extrapolation SIMEX [8] corrections to MR-Egger analysis where I^2^_GX_ estimates were below 0.9 (which would indicate the effect estimate is biased by 10% due to measurement error) [7]. This analysis was first restricted to ever smokers to capture the long-term effects of smoking/nicotine use (i.e., including former smokers who may have quit smoking due to developing a health issue), and then further restricted to current smokers only (where the data were available) to explore the potential effects of smoking cessation (i.e., recoverable effects). If a poor health outcome was found among current smokers but not ever smokers, it would indicate that health outcome may improve following smoking cessation. Restricting the analysis to former smokers, we can further explore this potential effect. Finally, the analysis was restricted to never smokers to explore the potential presence of pleiotropic pathways and bias due to population stratification.

**References**

1. Bowden J, Davey Smith G, Burgess S. Mendelian randomization with invalid instruments: effect estimation and bias detection through Egger regression. Int J Epidemiol. 2015;44(2):512-25. Epub 2015/06/08. doi: 10.1093/ije/dyv080. PubMed PMID: 26050253; PubMed Central PMCID: PMCPMC4469799.

2. Hartwig FP, Davey Smith G, Bowden J. Robust inference in summary data Mendelian randomization via the zero modal pleiotropy assumption. Int J Epidemiol. 2017;46(6):1985-98. Epub 2017/10/19. doi: 10.1093/ije/dyx102. PubMed PMID: 29040600; PubMed Central PMCID: PMCPMC5837715.

3. Burgess S, Butterworth A, Thompson SG. Mendelian randomization analysis with multiple genetic variants using summarized data. Genet Epidemiol. 2013;37(7):658-65. Epub 2013/10/12. doi: 10.1002/gepi.21758. PubMed PMID: 24114802; PubMed Central PMCID: PMCPMC4377079.

4. Bowden J, Davey Smith G, Haycock PC, Burgess S. Consistent Estimation in Mendelian Randomization with Some Invalid Instruments Using a Weighted Median Estimator. Genet Epidemiol. 2016;40(4):304-14. Epub 2016/04/12. doi: 10.1002/gepi.21965. PubMed PMID: 27061298; PubMed Central PMCID: PMCPMC4849733.

5. Davey Smith G, Hemani G. Mendelian randomization: genetic anchors for causal inference in epidemiological studies. Hum Mol Genet. 2014;23(R1):R89-98. Epub 2014/07/30. doi: 10.1093/hmg/ddu328. PubMed PMID: 25064373; PubMed Central PMCID: PMCPMC4170722.

6. Lawlor DA, Tilling K, Davey Smith G. Triangulation in aetiological epidemiology. Int J Epidemiol. 452016. p. 1866-86.

7. Bowden J, Del Greco MF, Minelli C, Davey Smith G, Sheehan NA, Thompson JR. Assessing the suitability of summary data for two-sample Mendelian randomization analyses using MR-Egger regression: the role of the I2 statistic. Int J Epidemiol. 2016;45(6):1961-74. Epub 2016/09/13. doi: 10.1093/ije/dyw220. PubMed PMID: 27616674; PubMed Central PMCID: PMCPMC5446088.

8. Lederer W, Küchenhoff H. A short Introduction to the SIMEX and MCSIMEX. R News. 2006;6:26-31.
