# Supplementary material for: Estimating the health impact of nicotine exposure by dissecting the effects of nicotine versus non-nicotine constituents of tobacco smoke: A multivariable Mendelian randomisation study
Source: PLoS Genet. 2024 Feb 9;20(2):e1011157. doi: 10.1371/journal.pgen.1011157 (PMC10883537; doi:10.1371/journal.pgen.1011157)
Supplement: S7 Note — (DOCX) [file pgen.1011157.s007.docx]

**S7 Note**

There are three main assumptions which underlie instrumental variable (IV) analyses which are applicable to MR analyse:

1. Relevance – the genetic variant(s) used as IVs must be associated with the exposure of interest.
2. Independence – the genetic variant(s) must not share any unmeasured cause with the outcome (e.g., via population stratification, collider bias, dynastic effects, or assortative mating).
3. Exclusion restriction – the genetic variant(s) must not affect the outcome except through its potential effect on the exposure of interest.
