# Supplementary figures and images for: Estimating the health impact of nicotine exposure by dissecting the effects of nicotine versus non-nicotine constituents of tobacco smoke: A multivariable Mendelian randomisation study
Source: PLoS Genet. 2024 Feb 9;20(2):e1011157. doi: 10.1371/journal.pgen.1011157 (PMC10883537; doi:10.1371/journal.pgen.1011157)

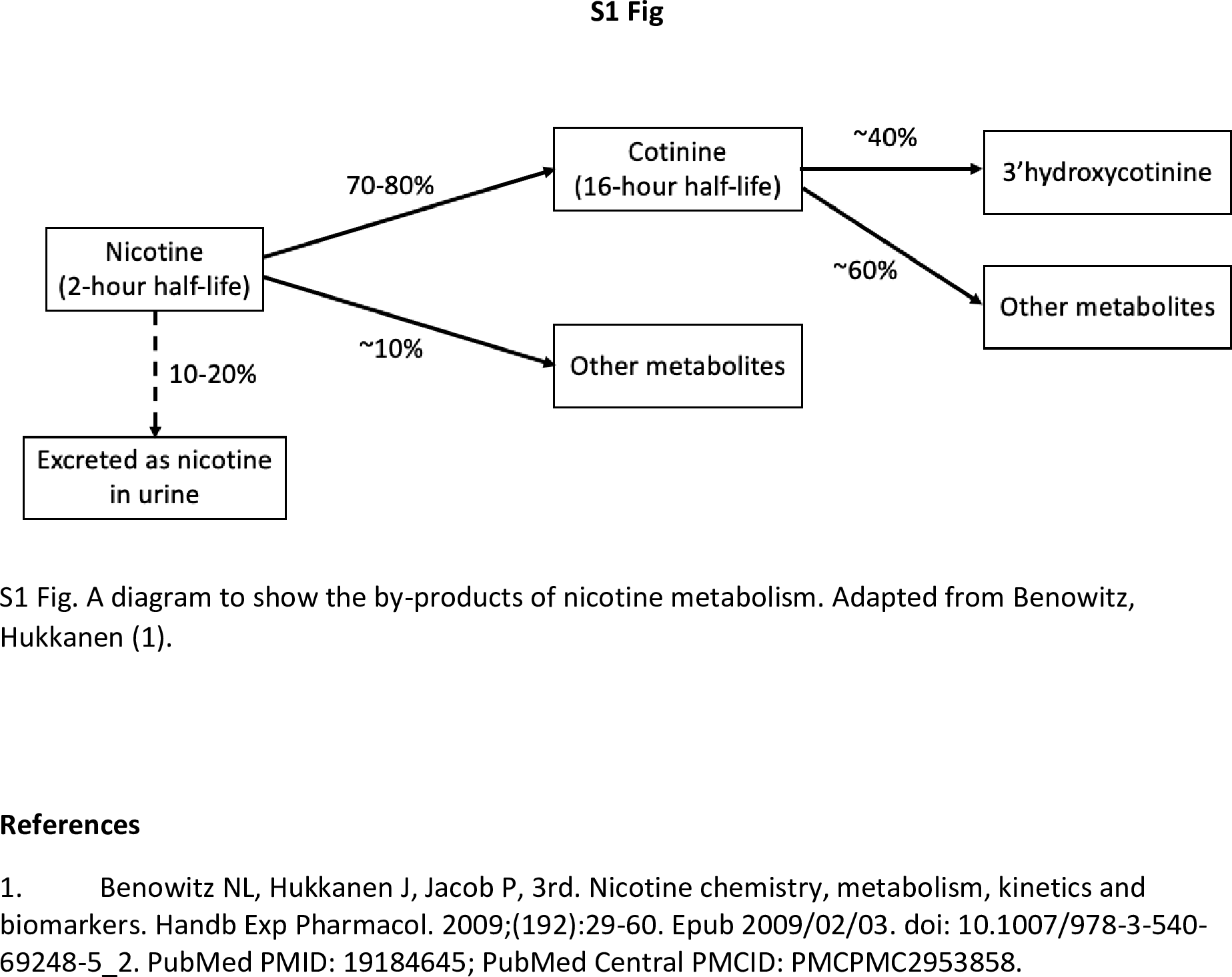

Supplement: S1 Fig — (TIF) [file pgen.1011157.s022.tif]

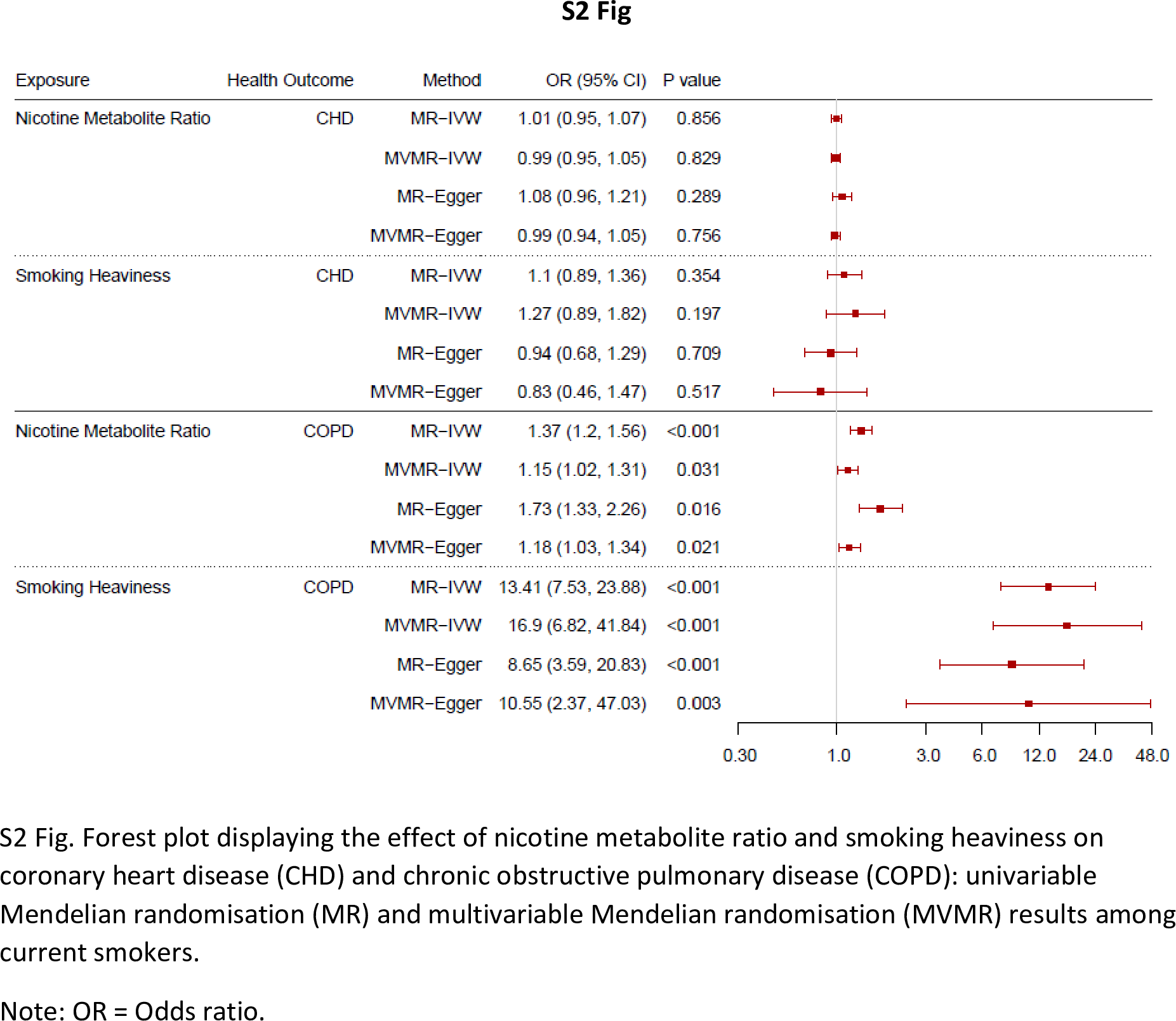

Supplement: S2 Fig — Note: OR = Odds ratio. (TIF) [file pgen.1011157.s023.tif]
