# Supplementary material for: Estimating the health impact of nicotine exposure by dissecting the effects of nicotine versus non-nicotine constituents of tobacco smoke: A multivariable Mendelian randomisation study
Source: PLoS Genet. 2024 Feb 9;20(2):e1011157. doi: 10.1371/journal.pgen.1011157 (PMC10883537; doi:10.1371/journal.pgen.1011157)
Supplement: Attachment — Submitted filename: Response to Reviewers_240111.docx [file pgen.1011157.s024.docx]

**Response to Reviewers**

*We thank the reviewers for their comments and have provided responses to each comment in italics below. Please note that when we refer to line numbers, these correspond to the document with highlighted changes.*

Reviewer #1:

1) While the analyses and results are well presented, the interpretation is completely irrelevant to the data that have been analyzed. There is no information about e-cigarettes and we have no direct information about smoking over time. The genetic instruments are proxies for behavior that may or may not happen and the low heritability of cigarettes per day mean that actual behaviors can have substantial impacts over the lifecourse that cannot be modeled in the way they have approached the analysis. That said, if the authors have access to the original UK Biobank data, since it is now a longitudinal study, and direct measures of smoking intensity along with the diseases they are studying are available, it would be possible to further analyze UKBB data to evaluate if the observations they are inferring from genetic proxies are reflected in the direct analyses that are possible in UKBB. Using UKBB as a primary source for these studies might not be optimal given limited numbers of lung cancers in this resource, but for validating findings such an analysis would be useful. On the other hand if the authors do not want to directly analyze smoking, nicotine and outcomes using direct multivariate studies, I think the paper has sufficient merit based on the analyses that have been completed to warrant consideration as a publication. However, the interpretation needs to be very carefully and almost completely rewritten.

*As the reviewer stated, genetic instruments proxy behaviour, and this behaviour may or may not occur. The genetic proxies used in this analysis were derived among smokers, and our main analyses were restricted to people who smoke, so the individuals have had first-hand cigarette smoke exposure. Although smoking heaviness is time-varying, MR estimates from time-varying exposures should be interpreted with respect to the underlying liability for an exposure [1, 2]. Therefore, the interpretation is the effect of the exposure on the outcome over the life course rather than at a specific time point. We have clarified this in the text on line 78:*

*“When using time-varying exposures in MR (e.g., smoking heaviness), MR results are interpreted as the effect of changing the liability of exposure at the time point measured [9, 10]. Assuming the genetic variants have a proportionally constant effect on the exposure across the lifetime, the MR effect estimate will be the genetically predicted lifetime effect of being a heavier smoker across the life course [11, 12].”*

*High heritability may increase the likelihood of identifying SNPs for a strong instrument, but high heritability is not necessary to ensure SNPs can be identified for a strong instrument. Although heritability is low in the case of smoking heaviness, the F-statistics indicate that we have strong instruments for the analyses conducted, therefore the low heritability is not an issue.*

*We appreciate Reviewer 1’s suggestion to analyse the observational data available in UK Biobank; however, the suggested multivariate analysis is not possible. UK Biobank does have data relating to smoking and our health outcomes, but there are no direct measures of nicotine or nicotine metabolites. Therefore, we would only be able to explore the association between cigarettes per day and our chosen outcomes. This would not add to our knowledge given that we selected outcomes known to be associated with heavier smoking.*

2) For example, the discussion from lines 538-551 is not supported by any of the data that have been analyzed, because the CHD proxies are constructed at birth based on genetic profiles and there is no information in this kind of analysis as it has been conducted to evaluate risk for CHD over the lifespan. One might theoretically construct genetic proxies for early and late age CHD and then that might give some inference about lifespan effects, but the authors have not done that so cannot make any speculative comments about lifecourse events from the analyses they have done.

*As lines 538-551 do not discuss CHD, we were unsure whether Reviewer 1 was referring to the paragraph before (lines 526-536), which discusses CHD, or if this was a typo and they meant to refer to CPD. We have provided some explanation of how MR results reflect life course risk of CPD exposure above (see response to comment 1) and clarified this in the manuscript (line 78). As CHD is an outcome, we do not look at the genetic risk of CHD directly, but look at the association of the genetic risk of the exposures with CHD up to the point of CHD measurement. We have also made amendments to the two discussion paragraphs as follows (line 560 onwards):*

*“In contrast to previous evidence, in which increased smoking heaviness was found to increase risk of CHD, we found limited evidence of an effect of smoking heaviness on CHD among ever smokers. Previous MR studies have also shown minimal evidence of this effect using UK Biobank data [24], which could indicate that the outcome GWAS sample may not be representative of the general population (i.e., selection or collider bias may have impacted our results). Indeed, UK Biobank participants are more likely to describe themselves as female, have fewer health issues, live in less socioeconomically deprived areas, and be more highly educated, than the general population and thus the results may suffer from “healthy volunteer” selection bias [46]. Given that CHD may disproportionately affect men [47] and people with generally poorer health, this may explain the limited findings. Additionally, we may have had limited power to detect this effect given the range of causes of CHD and relatively few cases in UK Biobank.*

*Another unexpected finding was the apparent protective effects of nicotine on lung health (COPD, FEV-1 and FVC) among current smokers. However, previous evidence suggests those with a lower NMR (and greater circulating nicotine per cigarette) inhale less smoke and other non-nicotine constituents per cigarette, and have higher levels of biomarkers of inflammation [19, 28]. Our MVMR model is designed to estimate nicotine exposure, but measuring smoking heaviness by the number of cigarettes smoked per day will not completely capture the variation in smoking intensity [48], which could complicate the interpretation of our findings. There could be a small effect of intensity which is captured in the instrumental variable for NMR because increased NMR may lead to increased inhalation per cigarette. Given the magnitude of effects found for NMR are much smaller than for smoking heaviness, the residual variance in smoking intensity impacting the NMR estimates appears to be limited. Furthermore, if there is a true protective effect it may not be clinically important given the magnitude of the effects found. For example, patients only perceive differences in FEV-1 with changes of 112 mL [49] whereas the MVMR-IVW analyses indicated an increase of only 17.77 mL per SD increase in NMR.”*

3) Similarly, there is absolutely no information in this study about e-cigarettes. Therefore, there is no relevance of any of the discussion about them. Aside from nicotine in e-cigarettes, there are many other compounds that do influence lung diseases. Therefore, all of the comments about risks from e-cigarettes are speculative and not tied to the analyses that were conducted. The All of Us cohort does collect data on e-cigarette use, so the authors could evaluate them from that cohort if they would choose to do that. Otherwise, they should remove all of the discussion of e-cigarette use in most of the paper. It may be possible to retain one sentence restricting commentary about e-cigarettes to just the impact that the nicotine component would have but the rest of the commentary needs to be removed to ensure consistency of the findings and their discussion with what was actually done in the analysis.

*We have removed some instances of discussion in relation to e-cigarette use; however, acknowledging that nicotine is now used without tobacco for extended periods is important for the rationale for the study. E-cigarettes which contain nicotine are frequently used for much longer periods than traditional nicotine replacement, and much of the evidence regarding the health effects of regular nicotine exposure is confounded by tobacco exposure. Since it is currently not possible to look at the health impact of e-cigarettes directly over the extended period required for many of these disease outcomes to emerge, we used MVMR to determine the health effects of nicotine (the main constituent of e-cigarettes. However, as highlighted by the reviewer, we are unable to draw conclusions on the other constituents of e-cigarettes. As we state on line 666: “further research is needed to understand the health impact of increased daily exposure to nicotine via other sources (e.g., e-cigarettes) which contain other components.”*

*The All of Us data set is an important resource that we are considering for further studies. However, there are a limited number of e-cigarette users in this data set and even fewer e-cigarette users who have never smoked – a restriction which is necessary given the potential shared genetic liability to smoking and vaping [3]. Furthermore, the long-term nature of the health outcomes we are investigating limits the investigation of vaping-related health effects in the All of Us data, whereas our model explores these outcomes among those with years or decades of exposure to nicotine via smoking (see response to comment 4).*

4) Starting with line 610 we again get an interpretation untied to any of the analyses that were done. The authors have modeled CPD and nicotine metabolism but then discuss long-term nicotine exposure via inhalation. If the authors had analyzed smoking duration or packyears of exposure with genetic proxies perhaps this paragraph would make some sense, but they analyzed CPD which is a one time measure unrelated to lifecourse. The entire paragraph should be removed. Similarly the discussion which describes many factors that were not studied needs to be completely rewritten. The kind of excessive extrapolations that are made in this manuscript can cause more harm than good in the literature if they are not significantly adjusted. While the ultimate message may be correct, the process taken to get to the message has to be accurately supported by analyses.

*As we discussed in our response to comment 1, MR estimates with time-varying exposures should be interpreted as an increase in exposure liability across the lifecourse (i.e. genetic liability to smoking heaviness). Although we could have used pack years or smoking duration as an exposure, this would have complicated our interpretation given that our model relies on the interplay between NMR and CPD (i.e., that we are accounting for nicotine exposure per cigarette smoked). Additionally, we decided to include a measure of smoking intensity rather than duration as this better aligns with the mechanism of NMR. However, we would like to highlight that the current smokers included in our UK Biobank GWAS had smoked a mean of 28 pack years (median = 25 pack years, interquartile range = 15 to 37 pack years), and less than 5% of the sample had less than 4 pack years of exposure. We have rewritten the discussion in response to the issues raised by the reviewer.*

*Line 603: “…however, the current smokers included in our UK Biobank GWAS had smoked a mean of 28 pack years (median = 25 pack years, interquartile range = 15 to 37 pack years), and less than 5% of the sample had less than 4 pack years of exposure.”*

*Line 654: “This work highlights the limited impact that increased daily nicotine exposure via smoking is likely to have on heart and lung health. However, further research is needed to understand the health impact of increased daily exposure to nicotine via other sources (e.g., e-cigarettes) which contain other components.”*

*Additionally, we have removed our reference to e-cigarette use in other places e.g.,: Line 594, “Although this is the first study to use MVMR to explore the impact of increased daily nicotine inhalation in humans …”, and we have instead referred to “regular” and “daily” nicotine use rather than long-term use which more accurately reflects the exposures measured.*

Reviewer #2: 

1) Abstract needs to be rewritten to increase clarity. For instance, more details are needed around GWAS used for the MV-MR analyses (data source, number of variants per trait, etc.). Also, line 28 starts with “these results suggest” although no results have been presented*.*

*We have revised the abstract:*

*“The detrimental health effects of smoking are well-known, but the impact of long-term nicotine use without exposure to the other constituents of tobacco is less clear. Given the increasing long-term use of alternative nicotine delivery systems, such as e-cigarettes, it is increasingly important to understand and separate the effects of long-term nicotine use from the impact of tobacco smoke exposure. Using a multivariable Mendelian randomisation framework, we explored the direct effects of nicotine compared with the non-nicotine constituents of tobacco smoke on health outcomes (lung cancer, chronic obstructive pulmonary disease [COPD], forced expiratory volume in one second [FEV-1], forced vital capacity [FVC], coronary heart disease [CHD], and heart rate [HR]). We used Genome-Wide Association Study (GWAS) summary statistics from Buchwald and colleagues, the GWAS and Sequencing Consortium of Alcohol and Nicotine, the International Lung Cancer Consortium, and UK Biobank*. *Increased nicotine metabolism increased the risk of COPD, lung cancer, and lung function in the univariable analysis. However, when accounting for smoking heaviness in the multivariable analysis, we found that increased nicotine metabolite ratio (indicative of decreased nicotine exposure per cigarette smoked) decreases heart rate (b = -0.30, 95% CI -0.50 to -0.10) and lung function (b = -33.33, 95% CI -41.76 to -24.90). There was no clear evidence of an effect on the remaining outcomes. The results suggest that these smoking-related outcomes are not due to nicotine exposure but are caused by the other components of tobacco smoke; however, there are multiple potential sources of bias, and the results should be triangulated using evidence from a range of methodologies.”*

*However, the number of traits per variant included in the analyses was different across analyses, so it would be unclear and too lengthy to add this to the abstract.*

2) Line 122: the exposure used in the MV-MR needs to be added.

*We have amended the introduction, now on line 151:*

*“We employed a two-sample MVMR framework to explore the direct effects of nicotine compared with the non-nicotine constituents of tobacco smoke (using genetic proxies for NMR and for CPD) on health outcomes (lung cancer, chronic obstructive pulmonary disease [COPD], forced expiratory volume in one second [FEV-1], forced vital capacity [FVC], coronary heart disease [CHD] and heart rate [HR]).”*

3) Lines 123-125: further rational on the outcomes explored needs to be provided.

*We have added a sentence before, and revised line 156-158:*

*“**These outcomes were selected as previous evidence has indicated they are causally impacted by smoking heaviness [1, 20-24]. The outcomes also include positive and negative control…”*

4) Line 136: A clarification on selection criteria for the variants used for each analysis needs to be added.

*A detailed description of the selection criteria is provided from line 794 under “selection of genetic variants” and further detailed in Supplementary Tables S9-S14 and Supplemental Note S5, as already discussed in the manuscript. We have added further clarification regarding conditional independence – see response to point 9.*

5) Line 143: A further discussion on sample overlap needs to be added. Exposure and outcome data are estimated in the UK Biobank and results obtained may be biased.

*Thank you for highlighting that we had mistakenly removed some information here. We have now added to line 715:*

*“To avoid sample overlap, two versions of the GSCAN data were used in our analyses. Where we analysed UK Biobank outcomes, we used a version of the GSCAN data with UK Biobank removed to avoid sample overlap. To maximise sample size, we used GSCAN data including UK Biobank in analyses which did not include UK Biobank in the outcome GWAS. In all analyses, 23andMe were removed from the GSCAN data due to data sharing restrictions.”*

6) Line 165: Betas and SEs for all analyses performed need to be added in supplementary material. It is not clear from the description of the methods what is number of variants used for each analysis.

*Supplementary Tables 9-14 now include information on the betas and standard errors for exposures used in each analysis. We have also added all data to an online repository (https://doi.org/10.5281/zenodo.10469666). The number of SNPs in each analyses were already included in these tables, but we have highlighted them to make them more noticeable. We have highlighted within the text (line 808 that the N for each analysis is in the supplementary material:*

*“Supplementary Tables S9-S14 detail which SNPs were included and excluded (with reasons**) and the total number of SNPs in each analysis. Supplemental Note S5 gives further details of the proxy search and clumping methods.”*

7) Line 215: Further clarification on the conversion of results obtained from BOLT to reflect Odds Ratios needs to be added. For instance, was prevalence of the outcome accounted for in the formula used?

*As stated in the UK Biobank pipeline documentation cited, the formula used is log𝑂𝑅 = 𝛽𝑏𝑜𝑙𝑡 / 𝜇 (1 – 𝜇), where 𝜇 = 𝑛𝑐𝑎𝑠𝑒 ⁄ (𝑛𝑐𝑎𝑠𝑒 + 𝑛𝑐𝑜𝑛𝑡𝑟𝑜𝑙) is the case prevalence. The standard errors are adjusted using the same transformation 𝑠𝑒(log𝑂𝑅) = 𝑠𝑒𝑏𝑜𝑙𝑡 / 𝜇(1 – 𝜇). We have amended the text to:*

*“binary phenotypes were transformed to log odds ratios and their corresponding 95% confidence intervals (95% CI) on the liability scale using a Taylor transformation expansion series in which we divided the statistics by 𝜇(1 – 𝜇), whereby 𝜇 is the case prevalence [4].”*

8) Line 213: Package used for the analyses need to be added as well as codes posted on a repository would be of help for the findings to be reproducible.

*Thank you for this suggestion, we have uploaded the code online and amended the text as follows: “All analyses were conducted using R version 4.1.1. The statistical analysis was completed using the TwoSampleMR, MVMR and MendelianRandomization packages, and the statistical code and data used is available online (https://doi.org/10.5281/zenodo.10469666).”*

9) Line 237: Please clarify what is meant by “conditional”.

*We have clarified this on lines 795-804:*

*“We selected conditionally independent (at the genome-wide significant level, p < 5 × 10-8) genetic variants identified in the conditional GWAS analyses of NMR and CPD for inclusion in the analysis [14, 15]; 55 SNPs associated with smoking heaviness were identified as conditionally independent [14]; 7 SNPs associated with the NMR were identified as conditionally independent. Methods of determining conditional independence varied between the two exposure GWAS, whereby GSCAN reported SNPs as independent if they explain additional variance in conditional analyses using a partial correlation-based score statistic [14] and Buchwald and colleagues reported SNPs as conditionally independent if they explained additional variance in a step-wise conditional regression using genome-wide complex trait analysis [15]”*

10) Line 243: How many SNPs where removed from each analysis?

*Each SNP removed / excluded from each analysis is identified in Supplementary Tables 9-14. We do not feel this needs repeating in the text given the number of analyses and length of the manuscript. We highlight in the next sentence where this information can be found:*

*“Supplementary Tables S9-S14 detail which SNPs were included and excluded (with reasons) and the total number of SNPs in each analysis. Supplemental Note S5 gives further details of the proxy search and clumping methods.”*

11) Line 260: Please add univariable MR to increase clarity.

*We have amended line 823: “For comparison with the MVMR analysis, we considered the total effect of both the NMR and smoking heaviness on each health outcome using univariable MR.”*

12) Line 306: Cochran’s Q statistics and p-values need to be added to show if heterogeneity is significant across various effect estimates.

*The Cochran’s q statistics are displayed in Supplementary Table S1 – S6. We have now indicated this in the text, line 338:*

*“Where the Cochran’s Q statistic (reported in Supplementary Tables S1 – S6) was greater than the number of SNPs…”*

13) Figure 3. A further explanation on why you observe a stronger association of smoking heaviness-COPD in the MV analyses compared to univariable analyses is needed.

*As the confidence intervals overlap, we cannot confidently say that there is a stronger association observed in this comparison. Also, the effect size is only greater in the IVW analysis, not the Egger analysis. To discuss this may be misleading.*

14) Figure 5. Please clarify “B” in the figure legend.

*We have added “Note: B = beta” to Figures 4 and 5, and “Note: OR = Odds ratio” to Figure 3 and Supplementary Figure S2.*

***References***

1.	Morris TT, Heron J, Sanderson ECM, Davey Smith G, Didelez V, Tilling K. Interpretation of Mendelian randomization using a single measure of an exposure that varies over time. International Journal of Epidemiology. 2022;51(6):1899-909. doi: 10.1093/ije/dyac136.

2.	Sanderson E, Richardson TG, Morris TT, Tilling K, Davey Smith G. Estimation of causal effects of a time-varying exposure at multiple time points through multivariable mendelian randomization. PLOS Genetics. 2022;18(7):e1010290. doi: 10.1371/journal.pgen.1010290.

3.	Khouja JN, Wootton RE, Taylor AE, Davey Smith G, Munafò MR. Association of genetic liability to smoking initiation with e-cigarette use in young adults. medRxiv. 2020:2020.06.10.20127464. doi: 10.1101/2020.06.10.20127464.

4.	Loh PR, Kichaev G, Gazal S, Schoech AP, Price AL. Mixed-model association for biobank-scale datasets. Nat Genet. 2018;50(7):906-8. doi: 10.1038/s41588-018-0144-6. PubMed PMID: WOS:000437224400002.

5.	Jiang Y, Chen S, McGuire D, Chen F, Liu M, Iacono WG, et al. Proper conditional analysis in the presence of missing data: Application to large scale meta-analysis of tobacco use phenotypes. PLOS Genetics. 2018;14(7):e1007452. doi: 10.1371/journal.pgen.1007452.

6.	Yang J, Lee SH, Goddard ME, Visscher PM. GCTA: a tool for genome-wide complex trait analysis. Am J Hum Genet. 2011;88(1):76-82. Epub 2010/12/17. doi: 10.1016/j.ajhg.2010.11.011. PubMed PMID: 21167468.
